# Endocrine toxicity of immune checkpoint inhibitors: essential crosstalk between endocrinologists and oncologists

**DOI:** 10.1002/cam4.1145

**Published:** 2017-07-18

**Authors:** Frédéric Illouz, Claire Briet, Lucie Cloix, Yannick Le Corre, Nathalie Baize, Thierry Urban, Ludovic Martin, Patrice Rodien

**Affiliations:** ^1^ Department of Endocrinology, Diabetes and Nutrition Reference Centre of Rare Thyroid Disease Hospital of Angers Angers Cedex 09 F‐49933 France; ^2^ MITOVASC Institute INSERM U1083 University of Angers Angers Cedex 09 F‐49933 France; ^3^ Department of Endocrinology Hospital of Orléans Orléans F‐45000 France; ^4^ Department of Dermatology Hospital of Angers University of Angers Angers Cedex 09 F‐49933 France; ^5^ UTTIOM Unit for Innovate Therapy in Medical Oncology Hospital of Angers Angers Cedex 09 F‐49933 France; ^6^ Department of Pulmonology and Thoracic Oncology Hospital of Angers Angers Cedex 09 F‐49933 France; ^7^ INSERM UMRS 1066 University of Angers Angers Cedex 09 F‐49933 France

**Keywords:** Adverse events, endocrine toxicity, immune checkpoint inhibitors, immunotherapy, thyroid dysfunction

## Abstract

Two types of immune checkpoint inhibitors, both antibodies that target cytotoxic T‐lymphocyte antigen‐4 and those that target programmed cell death‐protein 1, have been approved for use in melanoma, non‐small‐cell lung cancer, and renal cell carcinoma as first‐line or second‐line therapy. Their adverse events are primarily regarded as immune‐related adverse events. We felt it was important to pinpoint and discuss certain preconceptions or misconceptions regarding thyroid dysfunction, hypophysitis, and diabetes induced by immune checkpoint inhibitors. We have identified areas of uncertainty and unmet requirements, including essential interaction between endocrinologists and oncologists. Five issues have been identified for discussion: (1) diagnosis of endocrine toxicity, (2) assessment of toxicity severity, (3) treatment of toxicity, (4) withdrawal or continuation of immunotherapy, (5) preventive action.

## Introduction

Cancer immunotherapy using anticytotoxic anti‐T‐cell antigen‐4 (CTLA4) antibodies (anti‐CTLA4) or anti‐programmed cell death‐protein1 (PD1) antibodies (anti‐PD1) have been approved for melanoma, non‐small‐cell lung cancer, and renal cell carcinoma and are also under investigation with respect to various other cancer types. These antibodies are directed against inhibitory and costimulatory molecules and result in activation of the immune system, in order to enhance tumor immunity. Cancer immunotherapy has improved progression‐free survival and overall survival in these tumors 1, 2. However, by increasing inhibitory signals, these antibodies disrupt peripheral tolerance and induce activation of autoimmune lymphocytes. Thus, their adverse events are primarily regarded as immune‐related adverse events (irAEs). CTLA‐4 inhibition possibly activates a wide range of T cells in the lymphoid organs implicated in self‐tolerance. Anti‐PD1 target T cells more specifically in the tissues, which might explain the different frequencies of toxicity between anti‐CTLA4 and anti‐PD1 3.

Oncologists have started to address these new irAEs over the past 10 years, and much has been learnt in certain situations such as intestinal irAEs. Frequent toxicities directed against the endocrine glands target the pituitary gland, the thyroid, and the pancreas 4, 5, 6. We note that the management of these irAEs was not based on hard data, but instead seemed to be transposed from irAEs in other systems, for instance the gastrointestinal tract or the liver. Our aim is to underline some preconceptions or misconceptions surrounding endocrine toxicity.

## Diagnosis of Endocrine Toxicity

A review of randomized studies shows that ICPI may induce “endocrine toxicity” such as thyroid dysfunction, hypophysitis, or diabetes. However, information relating to these endocrine toxicities is often scarce and imprecise 7, 8, 9. Precise delineation of the natural history of these toxicities is a mandatory step toward understanding their physiopathological mechanisms and facilitating specific subsequent management of the dysfunction. The first example deals with trials reporting “thyroid dysfunction”. Hypothyroidism was reported in up to 10% of patients receiving monotherapy but could be more frequent (up to 25%) in sequential or combined ipilimumab, nivolumab, and pembrolizumab therapy 7, 10, 11, 12. Hyperthyroidism is less frequent but was reported in up to 5%, and in up to 9.9% of cases receiving combined ipilimumab and nivolumab therapy 8, 11. A recent study reported subclinical hyperthyroidism in 13% of patients receiving anti‐PD1, in 16% of patients receiving ipilimumab, and in 22.2% of patients receiving a combination of nivolumab and ipilimumab 12. However, simple reference to “hypothyroidism” and “thyrotoxicosis” in reports is insufficient to establish appropriate diagnosis or effective management of the toxicity 8, 13, 14. Only two reports indicated that thyroid dysfunction results from destructive thyroiditis in most cases 15, 16. The latter presents with an initial phase of thyrotoxicosis followed by long‐term (or definite) hypothyroidism, as may also be observed in treatment using tyrosine kinase inhibitors. Consequently the thyrotoxic phase is brief, thus questioning the necessity of treatment for transient toxicity. Significantly, several cases of euthyroid Graves's ophthalmopathy was described involving ipilimumab but with no connection to anti‐PD1 17. The molecular mechanisms underlying thyroid toxicities remain unclear, and the role of CTLA‐4 receptor gene polymorphic variants has been evoked since some variants conceivably increase the risk of thyroid dysfunction 18, 19, 20.

The laboratory tests used to monitor thyroid function raise significant concerns. TSH measurement is the routine basal test used to evaluate thyroid status. However, ICPI‐related hypophysitis has been reported, especially in ipilimumab therapy 21. TSH levels have a tendency to be, and as a rule are normal in central hypothyroidism as in cases of hypophysitis. Missed diagnosis of central hypothyroidism will occur if FT4 is not measured in addition to TSH. In some cases of hypophysitis, TSH levels may even be suppressed which will misleadingly result in diagnosis of hyperthyroidism in the absence of FT4 evaluation. In some patients, TSH levels in the upper limit of normal range with low FT4 levels suggest combined pituitary and thyroid failure. Moreover, high‐dose steroids used to treat pain or cerebral edema, can downregulate the thyrotropic axis and reduce TSH levels. These medications must be considered when interpreting TSH results. Nevertheless, two recent overviews omit recommending the diagnostic strategy consisting of distinguishing thyroid and pituitary dysfunction. 22, 23 Although thyroid dysfunctions are on the whole seemingly due to primary thyroid disorders, both TSH and FT4 levels must be measured in patients receiving ICPI to determine the type of thyroid axis abnormality. These precautions will enable evaluation of the true frequency of thyroid axis abnormalities and improve their management.

The second example refers to “adrenal insufficiency”. This toxicity was reported in 1% to 3% of patients. 11, 24 Although several reviews reported its frequency, it is clear that in trial reports, this term indiscriminately encompasses primary adrenal insufficiency (featuring glucocorticosteroid, androgen, and mineralocorticoid deficiency) and ACTH (adrenocorticotropic hormone) deficiency (limited to glucocorticosteroid and androgen deficiency) which may conceivably be due to bilateral adrenal metastasis or pituitary metastasis, respectively. Only one case report described the coexistence of adrenal insufficiency and pituitary corticotropin deficiency but hormonal blood tests are absent 25. Moreover, only one case report focused on primary adrenal insufficiency alone with glucocorticoid and mineralocorticoid function deficit 26. Insufficient information precludes affirmation of direct damage to the adrenal gland by ICPI 4. However, suppression of ACTH may also result from glucocorticosteroid therapy (the use of which is common during medical treatment of cancer), or ultimately constitute an immunotherapy‐related adverse event (hypophysitis) 7, 27, 28. Thus, before considering glucocorticosteroid insufficiency as an adverse event of ICPI, adrenal and pituitary imaging must be performed to exclude metastasis. Recent reviews have made no recommendations concerning screening and diagnosis of glucocorticoid insufficiency 5, 22, 24. When glucocorticoid insufficiency is suspected, cortisol and ACTH levels should be measured in the morning to confirm the etiology of this insufficiency. A cosyntropin stimulation test may be performed in the event of inconclusive basal measurements, but many factors can influence interpretation of this test (performance status, medications, early or late diagnosis). The cortisol cut‐off point will necessarily be determined according to each patient. An accurate investigation of these endocrine abnormalities is mandatory when reporting adverse events, in addition to the use of appropriate and precise terminology according to Common Terminology Criteria for Adverse Events (CTCAE). Thus, a dialog between the oncologist and the endocrinologist is necessary to precise the etiology of glucocorticoid insufficiency. This point is important since, in case of a primary adrenal, mineralocorticoid substitution will have to be associated with glucocorticoid substitution.

## Grading the Severity of Endocrine Toxicity

The use of CTCAE is recommended when grading the severity of adverse events resulting from all types of therapy, including cancer therapy. Endocrine adverse events, including severe events (≥grade 3), are described in ICPI treatment 8, 16. The grading is designed to help physicians decide whether to continue, adjust dosage, or discontinue ICPI. It also provides accurate information on when and how to treat adverse events. Grading is based on symptoms and the necessity of hospital admission. With reference to endocrine adverse events, it does not seem advisable to base action on clinical signs. Hypothyroidism‐ and thyrotoxicosis‐related symptoms are mostly nonspecific and frequently overlooked (in hypothyroidism: fatigue, constipation, cold intolerance, weight gain; in hyperthyroidism: diarrhea, heat intolerance, palpitation, tachycardia, atrial fibrillation, weight loss); hence, it is inappropriate to await complications of hypothyroidism or cardiothyrotoxic crisis before adjusting therapy. Likewise, glucocorticoid insufficiency is characterized by nonspecific symptoms such as weakness, nausea, abdominal pain, fever, and vomiting, which may be frequent in patients undergoing cancer therapy. Adrenal crisis is a life‐threatening event characterized by hypotension or hypovolemic shock, fever, vomiting, coma, and electrolyte imbalance. Early detection of glucocorticoid insufficiency before adrenal crisis is therefore mandatory in order to initiate immediate hormonal replacement. We believe that the CTCAE grading system is unsuitable for endocrine adverse events and that use thereof in its current form is possibly even dangerous 22, 23. If glucocorticosteroid insufficiency was detected before acute crisis onset, which entails appropriate screening, patient performance status would most certainly improve and morbidity/mortality decline.

It is difficult to discern whether hypophysitis constitutes a severe event. Unrecognized pituitary insufficiency can be life‐threatening due to corticotropic deficiency, whereas hormone replacement in acknowledged pituitary failure is uncomplicated. Only pituitary stalk enlargement associated with compression of the optic chiasm should, in our opinion, be considered a severe adverse event. For this reason pituitary MRI should be performed in the event of suspected hypophysitis 21.

Diabetes mellitus is now reported as an adverse event in various types of anti‐PD1 6, 29. The hallmark of this type of diabetes is rapid rise in blood glucose levels in insulinopenic mode. Cases of diabetic ketoacidosis have been reported 6, 29. Again, CTCAE grading based on glucose levels would appear to be inappropriate since moderately elevated glucose levels may point to fulminant diabetes, and their detection could be invaluable. Here again, the use of corticosteroids may be a precipitating factor. An endocrinologist should be consulted urgently so as to define an appropriate glucose monitoring schedule and determine the necessity for insulin administration. In order to prevent severe hyperglycemia, we recommend blood glucose monitoring prior to each injection of ICPI.

## Management of Endocrine Adverse Events

Because ICPI modulate immune responses, high‐dose corticosteroid treatment is proposed. This strategy is proposed in gastrointestinal or liver toxicity 23. To the best of our knowledge, there is no available data demonstrating the benefit of prednisone/prednisolone in endocrine toxicity. Corticosteroids have been recommended in certain cases of severe thyrotoxicosis, but data pertaining to the management of endocrine adverse events in large trials is scarce and limited. 8, 23, 30 *β*‐blockers seem adequate in managing the thyrotoxic phase of thyroiditis 15, 16. With respect to hypothyroidism, some authors followed the recommendations of trial sponsors and either initiated thyroxine replacement in the presence of clinical symptoms or proposed “specialist advice” 31, 32. Although levothyroxine was proposed in two recent reviews, recommendations pertaining to its introduction are lacking. 22, 23 We believe that hypothyroidism symptoms are so unspecific that their use in determining initiation of thyroid replacement would be dangerous. Instead, we propose introducing levothyroxine when TSH levels are > 10mUI/L as is usually recommended in adult primary hypothyroidism 33. With regard to central hypothyroidism, TSH levels are uninformative and unhelpful, as previously mentioned, and we propose the introduction of thyroxine when FT4 levels fall below the lower limit of normal range, regardless of TSH levels.

High‐dose corticosteroids are recommended in the treatment of hypophysitis 32, 34. However, it has recently been shown that high‐dose corticosteroids fail to improve both the resolution of pituitary enlargement and pituitary insufficiency 5, 35, 36. Moreover high‐dose corticosteroids are in fact able to induce corticotropic insufficiency. In the absence of pituitary enlargement, treatment should be restricted to hormone replacement. However, in the event of enlarged pituitary glands, high‐dose prednisone/prednisolone seems appropriate in limiting inflammation and preventing optic nerve compression 23. Such cases are more frequently ipilimumab‐related than anti‐PD1‐related 21.

## Should Immunotherapy be Withdrawn or Continued in Endocrine AE?

This question is of importance when ICPI treatment is effective. Surprisingly, the discontinuation or continuation of ICPI has not been systematically debated in recent reviews 5, 24, 34, 37, 38. Torino et al. propose discontinuation of ICPI in the event of grade 3 or 4 hypophysitis and Corsello et al. propose discussion of strategy on an individual basis 24, 34. In a recent review, the authors consider that resumption of ICPI is feasible in most cases of thyroid toxicity, although several reviews make no such recommendation 4, 5, 24, 34, 37, 38, 39. Except in cases of life‐threatening endocrine toxicity which are unsuitable for efficient treatment, the decision to continue ICPI should not be based on the presence of endocrine toxicity but rather on the risk involved in discontinuing therapy in patients under treatment for controlled or responsive malignant disease. Withdrawal would, in such cases, represent a loss of opportunity. Even in cases of life‐threatening endocrine toxicity (such as glucocorticoid insufficiency), discontinuation of therapy and reintroduction after recovery may be discussed collaboratively by oncologists and endocrinologists, as proposed by González‐Rodriguez et al. 39. Indeed, all cases of hormone insufficiency can be easily resolved by hormone replacement in accordance with the basic work of endocrinologists. This statement is supported by a recent study showing that occurrence of ipilimumab‐induced hypophysitis does not appear to alter patient prognosis 40.

## Preventive Action and Monitoring of Patients with ICPI

**Figure 1:**
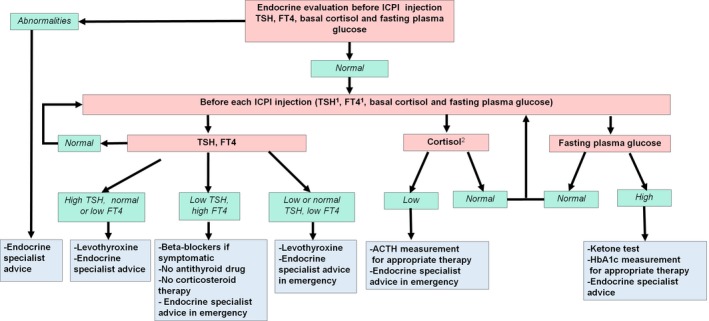
Proposition of endocrine test evaluation during ICPI therapy. ^1^ TSH and FT4 measurement could be performed every week during the first 2 months of ICPI; ^2^in the absence of corticosteroid treatment.

To avoid severe endocrine adverse events, the most effective strategy is necessarily a preventive one. In view of the scarcity of systematic prospective data on ICPI endocrine adverse events, in our opinion, no objective recommendations can be made. Either we can use current guidelines of package inserts of molecules or we can propose a new algorithm based on clinical experience and limited data. In our systematic hormonal blood test evaluation (data as yet unpublished), we observe highly precocious thyroid disorders after the first or second injection of ICPI. Central glucocorticoid insufficiency (due to hypophysitis) may also occur. Currently, ipilimumab package inserts merely recommend thyroid function test and ACTH evaluation before initial and each subsequent ipilimumab injection 41. Nivolumab and pembrolizumab package inserts are vague and propose thyroid function tests at the start of treatment, periodically during treatment, and as clinically indicated based on symptoms. Moreover, no specific screening guidelines and no blood tests are proposed to detect pituitary insufficiency 42, 43. Signs of endocrine disorders can remain undetected; hence, we believe that diagnosis of these toxicities relies exclusively on regular systematic hormonal evaluation in order to improve patient quality of life. We propose an algorithm (Fig. 1) to monitor ICPI endocrine toxicities but this can only serve as a basis for discussion and will necessarily be updated when new data occur. Methodical monitoring of thyroid dysfunction, glucocorticoid insufficiency, and glucose levels eliminates the risk of overlooking latent endocrine toxicity. As the adrenal glands, and less frequently the pituitary gland, can be metastatic sites, it is important that they be imaged before starting ICPI. Since hypopituitarism, thyroid dysfunction, and diabetes mellitus are the three major types of endocrine toxicity, we believe that TSH, FT4, cortisol, ACTH, and glucose measurement should be performed before initiating ICPI. Evaluation of other pituitary hormones is unwarranted because neither gonadotropic nor somatotropic insufficiency is life‐threatening. However, pituitary hormone evaluation could contribute to diagnosis of hypophysitis, even though its interpretation would necessarily involve several factors such as sex, age, medications, and performance status.

Objective recommendations for follow‐up are problematic because information on the timing of adverse event onset is often absent from large trials 7, 27, 32, 39, 44. The frequency of laboratory tests is not codified and ranges from no systematic screening to one single hormonal evaluation 8 weeks post initiation of ICPI therapy when there is no sign of endocrine toxicity 22, 23, 39, 45. However, some studies demonstrated that thyroid dysfunction may appear as early as the third week of therapy 15, 16. We therefore recommend thyroid function tests once per week during the first 2 months of ICPI and before each injection from the third month onward (Fig. 1). Cortisol and glucose levels should be evaluated before each injection of ICPI. Suspicion of endocrine dysfunction should prompt a new test. As previously mentioned, such recommendations will have to be adjusted according to our knowledge.

## Conclusion

Although certain endocrine adverse events are frequent during cancer immunotherapy, their management is relatively uncomplicated. A concerted approach by endocrinologists and oncologists is essential in order to determine the degree of severity of adverse events, establish the best course of treatment, and opt whether to continue or interrupt immunotherapy. In our view, clinical and biochemical screening of endocrine toxicity would improve our knowledge of physiopathological mechanisms as well as help modify our management and in preventing severe events, as is currently the case in toxicity induced by tyrosine kinase inhibitors. Our primary objective must remain the treatment of aggressive forms of cancer and the preservation of an acceptable quality of life for patients 46.

## Conflict of Interest

None declared.

## References

[1] Herzberg, B. , and D. E. Fisher . 2016 Metastatic melanoma and immunotherapy. Clin. Immunol. 172:105–110. 27430520 10.1016/j.clim.2016.07.006 PMC5569887

[2] Qin, A. , D. G. Coffey , E. H. Warren , and N. Ramnath . 2016 Mechanisms of immune evasion and current status of checkpoint inhibitors in non‐small cell lung cancer. Cancer Med. 5:2567–2578. 27416962 10.1002/cam4.819 PMC5055165

[3] Pardoll, D. 2012 The blockade of immune checkpoints in cancer immunotherapy. Nat. Rev. Cancer 12:252–264. 22437870 10.1038/nrc3239 PMC4856023

[4] Ryder, M. , M. Callahan , M. A. Postow , J. Wolchok , J. A. Fagin . 2014 Endocrine‐related adverse events following ipilimumab in patients with advanced melanoma: a comprehensive retrospective review from a single institution. Endocr. Relat. Cancer 21:371–381. 24610577 10.1530/ERC-13-0499 PMC4573438

[5] Faje, A. 2016 Immunotherapy and hypophysitis: clinical presentation, treatment, and biologic insights. Pituitary 19:82–92. 26186958 10.1007/s11102-015-0671-4

[6] Hughes, J. , N. Vudattu , M. Sznol , S. Gettinger , H. Kluger , B. Lupsa , et al. 2015 Precipitation of autoimmune diabetes with anti‐PD‐1 immunotherapy. Diabetes Care 38:e55–e57. 25805871 10.2337/dc14-2349 PMC4370325

[7] Weber, J. S. , G. Gibney , R. J. Sullivan , J. A. Sosman , C. L. Jr Slingluff , D. P. Lawrence , et al. 2016 Sequential administration of nivolumab and ipilimumab with a planned switch in patients with advanced melanoma (CheckMate 064): an open‐label, randomised, phase 2 trial. Lancet Oncol. 17:943–955. 27269740 10.1016/S1470-2045(16)30126-7 PMC5474305

[8] Larkin, J. , V. Chiarion‐Sileni , R. Gonzalez , J. J. Grob , C. L. Cowey , C. D. Lao , et al. 2015 Combined nivolumab and ipilimumab or monotherapy in untreated melanoma. N. Engl. J. Med. 373:23–34. 26027431 10.1056/NEJMoa1504030 PMC5698905

[9] Ribas, A. , R. Kefford , M. A. Marshall , C. J. Punt , J. B. Haanen , M. Marmol , et al. 2013 Phase III randomized clinical trial comparing tremelimumab with standard‐of‐care chemotherapy in patients with advanced melanoma. J. Clin. Oncol. 31:616–622. 23295794 10.1200/JCO.2012.44.6112 PMC4878048

[10] Postow, M. A. , J. Chesney , A. C. Pavlick , C. Robert , K. Grossmann , D. McDermott , et al. 2015 Nivolumab and ipilimumab versus ipilimumab in untreated melanoma. N. Engl. J. Med. 372:2006–2017. 25891304 10.1056/NEJMoa1414428 PMC5744258

[11] Ramelyte, E. , S. A. Schindler , and R. Dummer . 2017 The safety of anti PD‐1 therapeutics for the treatment of melanoma. Expert Opin. Drug Saf. 16:41–53. 27737598 10.1080/14740338.2016.1248402

[12] Morganstein, D. L. , Z. Lai , L. Spain , S. Diem , D. Levine , C. Mace , et al. 2017 Thyroid abnormalities following the use of cytotoxic T‐lymphocyte antigen‐4 and programmed death receptor protein‐1 inhibitors in the treatment of melanoma. Clin. Endocrinol. (Oxf) 86:614–620. 28028828 10.1111/cen.13297

[13] Ribas, A. , I. Puzanov , R. Dummer , D. Schadendorf , O. Hamid , C. Robert , et al. 2015 Pembrolizumab versus investigator‐choice chemotherapy for ipilimumab‐refractory melanoma (KEYNOTE‐002): a randomised, controlled, phase 2 trial. Lancet Oncol. 16:908–918. 26115796 10.1016/S1470-2045(15)00083-2 PMC9004487

[14] Gettinger, S. , N. A. Rizvi , L. Q. Chow , H. Borghaei , J. Brahmer , N. Ready , et al. 2016 Nivolumab monotherapy for first‐line treatment of advanced non‐small‐cell lung cancer. J. Clin. Oncol. 34:2980–2987. 27354485 10.1200/JCO.2016.66.9929 PMC5569692

[15] de Filette, J. , Y. Jansen , M. Schreuer , H. Everaert , B. Velkeniers , B. Neyns , et al. 2016 Incidence of thyroid‐related adverse events in melanoma patients treated with pembrolizumab. J. Clin. Endocrinol. Metab. 101:4431–4439. 27571185 10.1210/jc.2016-2300 PMC5095250

[16] Orlov, S. , F. Salari , L. Kashat , and P. G. Walfish . 2015 Induction of painless thyroiditis in patients receiving programmed death 1 receptor immunotherapy for metastatic malignancies. J. Clin. Endocrinol. Metab. 100:1738–1741. 25751110 10.1210/jc.2014-4560

[17] Min, L. , A. Vaidya , and C. Becker . 2011 Thyroid autoimmunity and ophthalmopathy related to melanoma biological therapy. Eur. J. Endocrinol. 164:303–307. 21088057 10.1530/EJE-10-0833 PMC4080629

[18] Donner, H. , J. Braun , C. Seidl , H. Rau , R. Finke , M. Ventz , et al. 1997 Codon 17 polymorphism of the cytotoxic T lymphocyte antigen 4 gene in Hashimoto's thyroiditis and Addison's disease. J. Clin. Endocrinol. Metab. 82:4130–4132. 9398726 10.1210/jcem.82.12.4406

[19] Donner, H. , H. Rau , P. G. Walfish , J. Braun , T. Siegmund , R. Finke , et al. 1997 CTLA4 alanine‐17 confers genetic susceptibility to Graves’ disease and to type 1 diabetes mellitus. J. Clin. Endocrinol. Metab. 82:143–146. 8989248 10.1210/jcem.82.1.3699

[20] Ueda, H. , J. M. Howson , L. Esposito , J. Heward , H. Snook , G. Chamberlain , et al. 2003 Gough SCAssociation of the T‐cell regulatory gene CTLA4 with susceptibility to autoimmune disease. Nature 423:506–511. 12724780 10.1038/nature01621

[21] Albarel, F. , C. Gaudy , F. Castinetti , T. Carré , I. Morange , B. Conte‐Devolx , et al. 2015 Long‐term follow‐up of ipilimumab‐induced hypophysitis, a common adverse event of the anti‐CTLA‐4 antibody in melanoma. Eur. J. Endocrinol. 172:195–204. 25416723 10.1530/EJE-14-0845

[22] Michot, J. M. , C. Bigenwald , S. Champiat , M. Collins , F. Carbonnel , S. Postel‐Vinay , et al. 2016 Immune‐related adverse events with immune checkpoint blockade: a comprehensive review. Eur. J. Cancer 54:139–148. 26765102 10.1016/j.ejca.2015.11.016

[23] Spain, L. , S. Diem , and J. Larkin . 2016 Management of toxicities of immune checkpoint inhibitors. Cancer Treat. Rev. 44:51–60. 26874776 10.1016/j.ctrv.2016.02.001

[24] Torino, F. , S. M. Corsello , and R. Salvatori . 2016 Endocrinological side‐effects of immune checkpoint inhibitors. Curr. Opin. Oncol. 28:278–287. 27136136 10.1097/CCO.0000000000000293

[25] Min, L. , and N. Ibrahim . 2013 Ipilimumab‐induced autoimmune adrenalitis. Lancet Diabetes Endocrinol. 1:e15. 24622375 10.1016/S2213-8587(13)70031-7 PMC4106239

[26] Trainer, H. , P. Hulse , C. E. Higham , P. Trainer , and P. Lorigan . 2016 Hyponatraemia secondary to nivolumab‐induced primary adrenal failure. Endocrinol Diabetes Metab Case Rep. [Epub ahead of print]. 10.1530/EDM-16-0108 PMC5097140 27857838

[27] Rizvi, N. A. , J. Mazières , D. Planchard , T. E. Stinchcombe , G. K. Dy , S. J. Antonia , et al. 2015 Activity and safety of nivolumab, an anti‐PD‐1 immune checkpoint inhibitor, for patients with advanced, refractory squamous non‐small‐cell lung cancer (CheckMate 063): a phase 2, single‐arm trial. Lancet Oncol. 16:257–265. 25704439 10.1016/S1470-2045(15)70054-9 PMC5726228

[28] Hodi, F. S. , S. J. O'Day , D. F. McDermott , R. W. Weber , J. A. Sosman , J. B. Haanen , et al. 2010 Improved survival with ipilimumab in patients with metastatic melanoma. N. Engl. J. Med. 363:711–723. 20525992 10.1056/NEJMoa1003466 PMC3549297

[29] Hofmann, L. , A. Forschner , C. Loquai , S. M. Goldinger , L. Zimmer , S. Ugurel , et al. 2016 Cutaneous, gastrointestinal, hepatic, endocrine, and renal side‐effects of anti‐PD‐1 therapy. Eur. J. Cancer 60:190–209. 27085692 10.1016/j.ejca.2016.02.025

[30] Gettinger, S. N. , L. Horn , L. Gandhi , D. R. Spigel , S. J. Antonia , N. A. Rizvi , et al. 2015 Overall survival and long‐term safety of nivolumab (anti‐programmed death 1 antibody, BMS‐936558, ONO‐4538) in patients with previously treated advanced non‐small‐cell lung cancer. J. Clin. Oncol. 33:2004–2012. 25897158 10.1200/JCO.2014.58.3708 PMC4672027

[31] Weber, J. S. , S. P. D'Angelo , D. Minor , F. S. Hodi , R. Gutzmer , B. Neyns , et al. 2015 Nivolumab versus chemotherapy in patients with advanced melanoma who progressed after anti‐CTLA‐4 treatment (CheckMate 037): a randomised, controlled, open‐label, phase 3 trial. Lancet Oncol. 16:375–384. 25795410 10.1016/S1470-2045(15)70076-8

[32] Robert, C. , G. V. Long , B. Brady , M. Maio , L. Mortier , J. C. Hassel , et al. 2015 Nivolumab in previously untreated melanoma without BRAF mutation. N. Engl. J. Med. 372:320–330. 25399552 10.1056/NEJMoa1412082

[33] Garber, J. R. , R. H. Cobin , H. Gharib , J. V. Hennessey , I. Klein , J. I. Mechanick , et al. ; American Association Of Clinical Endocrinologists And American Thyroid Association Taskforce On Hypothyroidism In Adults . 2012 Clinical practice guidelines for hypothyroidism in adults: cosponsored by the American Association of Clinical Endocrinologists and the American Thyroid Association. Thyroid 22:1200–1235. 22954017 10.1089/thy.2012.0205

[34] Corsello, S. M. , A. Barnabei , P. Marchetti , L. De Vecchis , R. Salvatori , and F. Torino . 2013 Endocrine side effects induced by immune checkpoint inhibitors. J. Clin. Endocrinol. Metab. 98:1361–1375. 23471977 10.1210/jc.2012-4075

[35] Min, L. , F. S. Hodi , A. Giobbie‐Hurder , P. A. Ott , J. J. Luke , H. Donahue , et al. 2015 Systemic high‐dose corticosteroid treatment does not improve the outcome of ipilimumab‐related hypophysitis: a retrospective cohort study. Clin. Cancer Res. 21:749–755. 25538262 10.1158/1078-0432.CCR-14-2353 PMC4334697

[36] Lammert, A. , H. J. Schneider , T. Bergmann , U. Benck , B. K. Krämer , R. Gärtner , et al. 2013 Hypophysitis caused by ipilimumab in cancer patients: hormone replacement or immunosuppressive therapy. Exp. Clin. Endocrinol. Diabetes 121:581–587. 24122241 10.1055/s-0033-1355337

[37] Abdel‐Rahman, O. , H. ElHalawani , and M. Fouad . 2016 Risk of endocrine complications in cancer patients treated with immune check point inhibitors: a meta‐analysis. Future Oncol. 12:413–425. 26775673 10.2217/fon.15.222

[38] Byun, D. J. , J. D. Wolchok , L. M. Rosenberg , and M. Girotra . 2017 Cancer immunotherapy—immune checkpoint blockade and associated endocrinopathies. Nat. Rev. Endocrinol. 13:195–207. 28106152 10.1038/nrendo.2016.205 PMC5629093

[39] González‐Rodríguez, E. , D. Rodríguez‐Abreu ; Spanish Group for Cancer Immuno‐Biotherapy (GETICA) 2016 Immune checkpoint inhibitors: review and management of endocrine adverse events. Oncologist 21:804–816. 27306911 10.1634/theoncologist.2015-0509 PMC4943391

[40] Faje, A. T. , R. Sullivan , D. Lawrence , N. A. Tritos , R. Fadden , A. Klibanski , et al. 2014 Ipilimumab‐induced hypophysitis: a detailed longitudinal analysis in a large cohort of patients with metastatic melanoma. J. Clin. Endocrinol. Metab. 99:4078–4085. 25078147 10.1210/jc.2014-2306

[41] http://packageinserts.bms.com/pi/pi_yervoy.pdf.

[42] http://packageinserts.bms.com/pi/pi_opdivo.pdf.

[43] https://www.merck.com/product/usa/pi_circulars/k/keytruda/keytruda_pi.pdf.

[44] Hodi, F. S. , J. Chesney , A. C. Pavlick , C. Robert , K. F. Grossmann , D. F. McDermott , et al. 2016 Combined nivolumab and ipilimumab versus ipilimumab alone in patients with advanced melanoma: 2‐year overall survival outcomes in a multicentre, randomised, controlled, phase 2 trial. Lancet Oncol. 17:1558–1568. 27622997 10.1016/S1470-2045(16)30366-7 PMC5630525

[45] Joshi, M. N. , B. C. Whitelaw , M. T. Palomar , Y. Wu , and P. V. Carroll . 2016 Immune checkpoint inhibitor‐related hypophysitis and endocrine dysfunction: clinical review. Clin. Endocrinol. (Oxf) 85:331–339. 26998595 10.1111/cen.13063

[46] Schadendorf, D. , R. Dummer , A. Hauschild , C. Robert , O. Hamid , A. Daud , et al. 2016 Health‐related quality of life in the randomised KEYNOTE‐002 study of pembrolizumab versus chemotherapy in patients with ipilimumab‐refractory melanoma. Eur. J. Cancer 67:46–54. 27596353 10.1016/j.ejca.2016.07.018

